# Post-diagnosis dementia care in the Western Pacific region: assessment of needs and pathways to optimal care

**DOI:** 10.1016/j.lanwpc.2024.101182

**Published:** 2024-09-16

**Authors:** Yun-Hee Jeon, David Foxe, Guk-Hee Suh, Huali Wang, Jacqueline C. Dominguez, Rex Maukera, Sengchanh Kounnavong, Olivier Piguet

**Affiliations:** a The University of Sydney, Susan Wakil School of Nursing and Midwifery, Faculty of Medicine and Health, Sydney, Australia; b The University of Sydney, School of Psychology and Brain and Mind Centre, Sydney, Australia; c Hallym University College of Medicine, Dept of Psychiatry, Seoul, South Korea; d Dementia Care and Research Center, Peking University Institute of Mental Health, PR China; e Institute for Neurosciences, St. Luke's Medical Center, Quezon City, Philippines; f National Psychiatric Unit, Kilu'ufi Hospital, Solomon Islands; g The Lao Tropical and Public Health Institute, Ministry of Health, Lao PDR

**Keywords:** Post-diagnosis care, Dementia, Rehabilitation, Western-Pacific region, Dementia care access

## Abstract

The Western Pacific region is home to approximately 25% of the world's population. In the absence of cures for dementia, it is essential to focus on appropriate and accessible care pathways for people living with dementia and their families. This approach will ultimately result in timely diagnosis and improved care and support.

Ensuring adequate dementia care and support pathways has been a longstanding issue in many developed countries and is becoming a more prominent issue in countries with rising dementia prevalence rates but comparatively limited health resources.

This Viewpoint provides an overview of system-level post-diagnosis dementia care, from diagnosis to rehabilitation, across some of the region's lower (Laos, Solomon Islands, Philippines) and upper (China) middle income and high income (South Korea, Australia) countries. Gaps and challenges in post-diagnosis dementia care, as well as suggestions for optimal care, are discussed. This Viewpoint highlights highly variable system level post-diagnosis dementia care in the region.

## Introduction

Dementia stands as one of the most significant global health challenges, ranking as the seventh leading cause of death worldwide and the second leading cause of death in developed countries. With the ageing of the world's population, the number of people living with dementia is expected to grow from 55 million to 139 million in the next 25 years. Such growth carries profound financial implications, with the estimated cost of dementia care surpassing USD$1.3 trillion in 2019, and with a projected increase to USD$2.8 trillion in less than six years.^1^

The Western Pacific region comprises 37 nations of low-, middle- and high-income countries.^2^ It is home to approximately 25% of the world's population with highly variable access to healthcare services. Over 60% (∼35 million) of the people living with dementia worldwide reside in low- and middle-income countries.^3^ In the absence of easily accessible disease-modifying treatments or cures, the focus needs to be on appropriate and accessible care services for people living with dementia and their families. Ensuring timely diagnosis and adequate post-diagnosis dementia care and support has been a longstanding issue in many developed countries. Equity of access to post-diagnosis care is likely to become a pressing issue in countries with rising dementia prevalence rates and comparatively limited health resources.

Post-diagnosis care and support (‘care’ hereafter) includes formal and informal care and support services that aim to optimise the health and well-being of people with dementia and their carers after diagnosis.^4^ These services vary in shapes and forms in terms of modes of delivery (e.g., episodic or ongoing), informal support by family or friends, location (e.g., hospital, outreach, community services, and virtual), as well as (non-)pharmacological treatments and interventions. The latest systematic reviews have shown the strong benefits that behavioural, cognitive, environmental, psychological and psychosocial interventions provide for people living with dementia and their family/informal carers.^5^ These findings are reflected in the recent international guidelines, which recommend physical exercise, cognitive behavioural therapy, cognitive stimulation therapy, and cognitive training for health and wellbeing of people with dementia.^6^ For family carers of people with dementia, mindfulness-based interventions, multicomponent interventions, psychoeducation, and psychotherapy/counselling are also recommended.^6^ However, what constitutes post-diagnosis care in formal services is highly variable.

For the purpose of this Viewpoint, we have limited post-diagnosis dementia care to services in the period following a dementia diagnosis, ideally immediately after, including services for pharmacological and non-pharmacological therapies, support for daily function and activities (cognitive, social and physical), management of behaviours and psychological symptoms, psychosocial services and rehabilitation/reablement, and support for family carers.^7^, ^8^, ^9^

Importantly, most research on post-diagnostic support has been predominantly conducted in and published by high-income countries, in particular in the Western Pacific Region. The aim of this Viewpoint is to fill this gap by providing an overview of post-diagnosis support services, and a lack thereof, of selected countries contrasting low-, middle- and high-income countries in the region, with additional contextual information about each country's population and health profile.

**Fig. 1:**
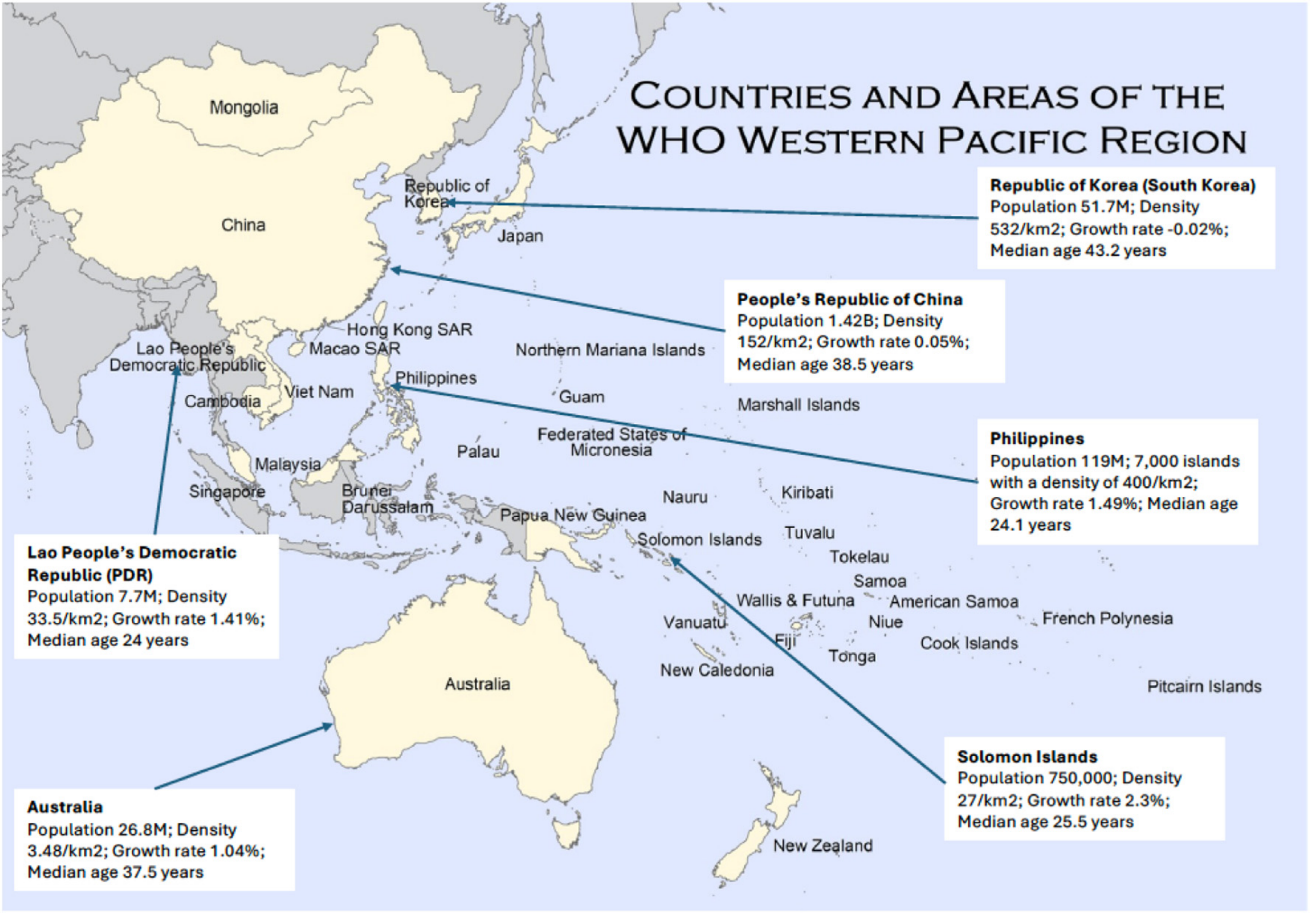
Countries and Areas of the WHO Western Pacific Region and Six Countries' Features. Note: Adapted from WHO Western Pacific Map. Countries and Areas of the Western Pacific Region at the following URL reference: https://www.who.int/westernpacific/about/where-we-work. The inserted textboxes for six countries contain information sourced from World Population Review https://worldpopulationreview.com. Accessed 4 June 2024. WHO is not responsible for the content or accuracy of this adaptation.

We illustrate this by examples contrasting lower middle (Lao People's Democratic Republic, Solomon Islands, the Philippines), upper middle (People's Republic of China) and high income (South Korea, Australia) countries.^10^ We purposively considered the countries' economic developmental status and determined the inclusion to balance the number from each band of low, middle and high income. For example, among high income countries such as Australia, Japan, New Zealand, Singapore and South Korea, only Australia, representing Oceania, and South Korea representing Asia, were selected. Experts (authors) from an initial list of countries were selected based on their standing in the field as well as their publication record and approached for their participation in the Viewpoint. Final decision was made taking into account availability of experts and literature from those low- and middle-income countries. No low-income country expert could be located during the search processes. Fig. 1 provides a graphic illustration of the six countries' locations and key characteristics.^2^

This Viewpoint presents an overview of system-level health and care services and care pathways for people with dementia and their family carers from the point of the dementia diagnosis to post-diagnosis care in selected countries with varying economic developments in the Western-Pacific region, identify some of the common and unique gaps in service provision and challenges that exist in the region, and provide suggestions for optimal post-diagnosis dementia care.

## Overview of system level post-diagnosis dementia care and services

### Lower middle-income countries

#### Lao People's Democratic Republic (PDR)

Lao PDR is a relatively young country, with only 4% of the population aged 65 years and over. Similar to other Southeast Asian countries, population growth has decreased markedly over the past 20 years, from 4.0% in 2005 to 0.2% in 2015.^11^

The health system in Lao PDR is currently going through an epidemiological transition, due to an ageing population, and socio-demographic and lifestyle changes. Previously led by communicable diseases, burden of disease is becoming dominated by non-communicable diseases, which are now the leading causes of morbidity and mortality.^12^^,^^13^ The prevalence of dementia in Lao PDR is not known. Recent data on 2200 individuals aged 60–98 years from the central, southern, and northern parts of the country showed that over 50% had cognitive deficits.^14^, ^15^, ^16^ These data, however, may need to be interpreted with caution in the light of the tool used and related cutoffs for cognitive deficits.^14^, ^15^, ^16^

A strong focus exists in the Lao PDR government on the welfare of older people in recognition of their contributions to the country. This position has been evident for over 20 years, with a number of reports outlining health promotion,^17^, ^18^, ^19^ in particular the National Policy Towards the Elderly of Lao PDR in 2004.^20^

No dementia national peak body currently exists in Lao PDR. The Elderly Association, which provides free health examination for its members twice a year and social and cultural activities promoting physical and mental health,^21^ is the only national peak body for older adults. The public sector provides the majority of inpatient and outpatient services. Health services, including preventive, educational and treatment services, have increased in recent years. Not surprisingly, marked differences across provinces, depending on economic status, remain in terms of the incidence of reported health issues and the care seeking behaviour.^22^

Perception and knowledge of dementia in Lao PDR remains limited, with dementia being commonly seen as a part of ‘normal ageing’. Recent surveys of community and official government services suggest that cognitive issues are considered less important than physical and mental changes.^23^ This position is reflected by the absence of government supported long-term care programs and facilities or dementia specific or geriatric facilities.

Hospital-based psychiatric units are responsible for providing assessments, treatments, and care plans for all adults, including dementia patients. No specific practice guidelines exist for dementia care and most older patients are cared for in the general ward for their physical, mental, and cognitive problems. Patients who are suspected of or diagnosed with dementia may be referred to a neurology ward or psychiatric ward at one of the central-referral hospitals in Laos, although no formal care pathway exists. Advanced neuroimaging tests, including CT and MRI, are offered only at the national referral hospital. In addition to diagnostic services, these central-referral hospitals provide education to families about dementia with a goal to strengthen family support for the patients (Personal Communication).

No national or local dementia-specific plan exists. Local action plans, however, have been in place for the past 15 years to ensure that the basic rights of all mentally ill individuals, including those with dementia, are respected.^24^ Preventive health measures, early diagnosis, and management of diseases are seen as health priorities by the Ministry of Health. Implementation of these priorities with regards to dementia has recently started in the two central-referral hospitals to record all dementia and neurological impairment patients for follow-ups and post-diagnosis care (Personal Communication).

#### Solomon Islands

The Solomon Islands are a chain of island provinces that are geographically dispersed. Three quarters of the population live in rural areas. The capital and largest city is Honiara (population: ∼67,000) with no other cities having a population over 10,000.^25^ Life expectancy has risen from 57 years in 1971 to 73.1 years in 2019.^25^^,^^26^ Dementia and Alzheimer's disease rank as the 20th most common cause of death in the Solomon Islands, accounting for 0.70% of all deaths.^27^

The Solomon Islands have integrated primary health services in the communities across the islands.^28^^,^^29^ This system is supported by secondary and tertiary services that are located in the provincial urban towns and in the capital Honiara,^29^ which serve ∼25% of the population.^25^ These services (Provincial Hospitals) have general Medical Officers and with specialists being found in the National Referral Hospital. Most of the Primary Health Services are run by registered nurses. These nurses, however, often have limited knowledge and skills in the care of people with dementia and providing support for their families through counselling and education.^29^

No post-diagnosis dementia-specific programs or services currently exist in the Solomon Islands. Care and support are generally provided by a family and relative network who often has close relationship with the person with dementia. Dementia is not recognised as a health priority and not seen as a public health concern. Health literacy in the general population is low and the availability of information about dementia is limited. In rural communities, people with dementia access Rural Health Facilities for any care and support like any other conditions and, in most cases, are not referred for specialist care. Cultural, religious and spiritual beliefs about dementia, combined with low literacy, are additional factors preventing access to services.^30^

Other barriers to appropriate health services exist for those living in the smaller, rural communities. Dementia is often perceived as a disease of old age, or as a curse for breaking certain cultural rules (e.g., committing a crime), or reflecting the loss of spiritual power the person possessed in the past. As such, dementia is commonly perceived as a mental health problem, which is generally managed at home. Patients seen in clinics or admitted to official health services are those exhibiting behavioural issues that cannot be supported at home or are in the later stages of dementia.^30^

Clinical diagnosis of dementia is confirmed at provincial hospitals, while presumptive diagnoses occur at local clinic levels. In the secondary and tertiary health services, dementia cases are referred to Psychiatric or Medical clinics, although no formal care pathways exist. A formal, evidence-based diagnosis is only possible in the National Referral Hospital and Psychiatric clinics. In addition to diagnostic services, these secondary and tertiary services provide education to families about dementia with a goal to strengthen family support for the client. Changed behaviours are primarily managed pharmacologically.

The progressive ageing of the population and increased life expectancy have resulted in an increase in the number of identified dementia cases, primarily in Honiara. The geographical dispersion of the population, not surprisingly, is creating logistical challenges with regards to referrals and management of people with dementia. Nevertheless, most cases are seen at first point entry locally and are rarely referred to specialist services.

Logistical, geographic, educational, and cultural/belief factors compound the challenge of providing dementia-relevant services in the Solomon Islands.

#### The Philippines

The elderly population at risk for dementia in the Philippines is estimated to reach 13.91 million by 2030.^31^ Currently subsumed under the ‘Mental Health’ program of the Department of Health, dementia is not currently a priority mental disorder.^32^ As such, funding for dementia care competes with other health priorities. Policies and plans primarily focus on other mental health disorders. The Philippine Mental Health program spends approximately USD$0.50 per capita for mental health, primarily used to fund mental hospitals and medicines.^32^

Dementia is generally perceived as a disease ‘natural’ to aging and a life changing experience that makes a person dependent on family members.^33^ A dementia diagnosis is usually prompted by the family and made by private specialists (e.g., neurologist, geriatrician, and psychiatrist), or general practitioners based on clinical examination, cognitive testing, and blood tests to assess vascular and metabolic risks.^34^ While brain imaging is recommended, it is not often pursued due to its high cost and limited accessibility.

Some dementia-related care is covered by the national health insurance *PhilHealth* at a case rate of up to USD$180 per person. However, this does not cover outpatient services where most dementia care is needed.^35^ No Department of Health-recommended dementia care pathway exists. The decentralised healthcare system, with multiple government agencies providing services, contributes to the fragmentation of dementia care in the Philippines.

In the absence of dementia-specific support services in the community, post-diagnostic care is led by the physician. Appropriate disclosure of diagnosis, caregiver education, referral for non-pharmacologic interventions, and long-term planning are offered *ad hoc*. Limited time in busy clinics, lack of trained staff, stigma, indifferent attitudes, and the unavailability of services all contribute to suboptimal post-diagnosis care. Overall, post-diagnosis care services are lacking except in few outpatient dementia clinics that formally provide care services.^36^ Due to limited resources, all care including respite and psychological care is almost exclusively provided in the form of informal care within family networks.^37^

The country has undertaken a number of dementia-related initiatives, based on the WHO Global Action Plan for Dementia (2017–2025).^3^ Presidential Proclamation No. 1136, s. 2006 is a broad initiative that stipulates the pursuit of strategies to contain dementia, reduce disability and economic burden.^38^ Through the Mental Health Act in 2018 and with active participation of non-government organisations such as Philippine Neurological Association and the Dementia Society of the Philippines, a national dementia plan will finally be included by the Philippine Council for Mental Health (PCMH) in its Strategic Plan 2024–2028.^39^

Further, the Department of Health is currently establishing geriatric centres that will improve early detection of dementia in regional hospitals. The Filipino translation of the WHO Dementia toolkit for low-and middle-income countries guides community workers in dementia care.^40^ Local practice guidelines for the diagnosis and treatment of dementia and care pathways guide healthcare professionals.^41^

Alzheimer's Disease Awareness Week is celebrated during the third week of September. The National Institute of Ageing disseminates Information, Education, and Communication (IEC) materials on dementia awareness via their website. The Alzheimer's Disease Association of the Philippines website has caregiver support materials and a list of care providers/care centres. A national government-funded program specific for dementia caregivers is yet to be legislated and implemented.^41^

### Upper middle-income country

#### People's Republic of China

In the People's Republic of China, most people seem to be able to recognise common indicators of dementia such as memory loss. In over two-thirds of cases, however, it takes at least 1 year for families to seek healthcare support.^42^ Despite a widespread understanding of the dementia symptoms among the general population, misconceptions regarding the appropriate care pathway persist.^43^

The National Health Commission officially announced a nationwide initiative known as the “2023–2025 Promotion Action Plan” (PAP) aimed at enhancing dementia care and prevention. This strategic endeavour is being spearheaded by a coalition of key organisations, including the Aging Health Department within the National Health Commission, the Center for Chronic Diseases at the National Center for Disease Control, and the Dementia Care and Research Center housed within Peking University's Institute of Mental Health.^44^ Alzheimer's Disease Chinese (ADC) stands out as the main peak body dedicated to dementia care. Additionally, numerous regional associations participate actively in advancing dementia care initiatives across the country.

Most people living with dementia receive home-based care under family support, with healthcare professionals driving diagnosis and treatment. Community-level efforts focus on prevention and early intervention by providing health education and screenings to reduce the risk of dementia. Hospitals primarily offer outpatient services, particularly in geriatric specialties, to improve timely diagnosis and treatment. There is an emphasis on culturally appropriate individualised interventions (education, prevention, risk management), enhanced professional and caregiver training, and adopting a multidisciplinary approach.^45^

Regional disparities in access to dementia care access are somewhat expected given the country's vast size, uneven distribution of resources, and varying levels of economic development. Economically advancing provinces like Zhejiang have well-integrated dementia care into routine aged care services. Dementia care resources are mostly concentrated in urban areas, and the dementia care strategic plan is still in its infancy in less developed regions.^46^

Memory clinics are increasingly common, and patients usually access hospitals for diagnosis. In regions where such services are lacking, diagnosis of dementia is made by geriatric neurology, psychiatry, and geriatric medicine specialists. The People's Republic of China's National Health Commission has established dementia-specific diagnostic and treatment guidelines, outlining the overall management approach.^47^

The government encourages broader participation from social organisations, often supported by local governments and partly by philanthropic investments, to support post-diagnosis services. Several significant gaps exist in post-diagnosis services and knowledge transfer. The services provided by social organisations need improvements in standardisation, monitoring, and outcome assessment. Through the PAP, standardised training is being implemented to develop skilled professionals, and initial evaluations are underway for care provided by social entities to enhance overall quality. The integration of medical and social services remains suboptimal.

### High income countries

#### South Korea

In 2023, 9.5 million people were aged 65 years and older (comprising 18.4% of the population), with 960,000 of them living with dementia.^48^ About 95% of the Korean population is covered by compulsory universal public health insurance, financed by premiums paid by beneficiaries, while the remaining 5% are covered by taxes.

Dementia has been recognised as a significant public health issue in South Korea. In 2012, the Dementia Management Act was enacted, and the 1st Comprehensive Dementia Management Plan (CDMP) began to build robust infrastructures for the National Dementia Responsibility System (launched in 2017).

As part of the 1st CDMP, nationwide public centres were established (1 central, 27 regional and 256 local dementia centres). These centres serve as a system for dementia prevention, early detection, expansion of treatment, and referral to dementia clinics and institutions across the country. The central dementia centre acts as a control tower for the National Dementia Management Project, while 27 regional dementia centres provide region-specific management and 256 local dementia centres provide dementia screening and referrals (i.e., visit dementia specialist clinics), management of people in the early stages of dementia, including symptom management, and emotional support for families of people with dementia. Dementia Counselling Call Centres also operate 24 h a day, 7 days a week.^49^ The 4th CDMP is currently underway.

Since the introduction of the long-term care insurance scheme in 2008, increasing national and local campaigns have provided public education about dementia and stigma reduction, supported by public health organisations, academic institutions, and media. While dementia awareness has greatly improved, stigma remains as one of the barriers to seeking diagnostic evaluation, community services, and support from family and friends.^50^

Dementia care is covered by long-term care and health insurances.^50^ The care level of individuals with dementia is determined by an independent long-term care committee, and about one in four people with dementia received their recommended care service in 2021. People with dementia are cared for in nursing homes and long-term care facilities (e.g. geriatric hospitals), mostly privately owned. Geriatric hospitals as well as in community care centres offer services such as home visits, day and night care, and respite care.^51^

Early detection of dementia has greatly increased since 2012. Free cognitive tests for early detection of dementia are offered every two years to people aged 60 or above at 256 local dementia centres. People at risk are referred to dementia specialist clinics led by psychiatrists or neurologists to confirm the dementia diagnosis. There, patients undergo various investigations (cognitive, blood, neuroimaging) to establish a differential diagnosis. It is estimated that more than half of people with cognitive impairment directly visit dementia specialist clinics.^52^ Upon dementia diagnosis, specialists provide management advice, including medications, and recommend appropriate community or institutional care services. To be eligible for relevant care services, the long-term care insurance scheme reviews and endorses the care level. Health professionals at each step throughout the dementia care pathway provide continuous services.

#### Australia

Dementia is the second leading cause of death in Australia and the leading cause among women.^53^ It is estimated over 400,000 Australians are living with dementia, with this number expected to double by 2058.^53^ The socioeconomic burden is projected to exceed $26.6 billion in the next two decades, representing an eightfold increase.^54^

The National Framework for Action on Dementia 2015–2019^55^ laid important groundwork, and a new National Dementia Action Plan^56^ spanning a decade is currently in development, scheduled for release in 2024.

Dementia care in Australia operates collaboratively between government and non-government organisations. At the federal level, the Australian government, primarily through the Department of Health and Aged Care, allocates funding for dementia care initiatives. Australian residents with dementia can access support through the National Disability Insurance Scheme (NDIS)^57^ for those younger than the age of 65 and through My Aged Care (MAC)^58^ for those 65 or older. NDIS and MAC coordinate access to subsidised services based on an individual's needs, determined through a care assessment. Key federally funded national services for dementia include the Dementia Behaviour Management Advisory Service (DBMAS) and Severe Behaviour Response Teams (SBRT) for identification, management and support for changed behaviours of people living with dementia.^59^ However, the approach to dementia care in Australia is mostly decentralised, with *hands-on* care embedded within state and territory health services. Each state/territory has its own initiatives, often coordinated by state/territory health departments or relevant authorities. Private, not-for-profit providers, and state public health services all play key roles in delivering services: coordination of these is necessary for wholistic care. Dementia Australia,^60^ a national peak body and advocacy entity for dementia, stands as the largest national resource. It is instrumental in providing comprehensive educational resources, counselling and support, and coordination for individuals and families impacted by dementia.

The diagnosis of dementia involves a multi-step process, starting with an initial assessment by a general practitioner (GP) who refers individuals with suspected dementia to relevant specialists (e.g., geriatrician, neurologist) or memory clinics for comprehensive evaluation. Following a diagnosis of dementia, a care plan is developed by the treating specialist or GP, outlining recommended interventions and support services. Individuals and their families are connected with support services, including public, private, and/or not-for-profit organisations. The care pathway is designed to be person-centred and collaborative, recognising the unique needs of individuals with dementia and their caregivers. Post-diagnosis care pathways in Australia, however, often lack standardisation, with variations in reviews and access to allied health services.^61^

Despite these positive developments, challenges persist. A significant disparity in access to information and services exists within Australia. Australia is a culturally and linguistically diverse nation.^62^ However, culturally and linguistically appropriate information remains limited, not only at the initial diagnosis consultation but also in post-diagnosis care and intervention.^62^, ^63^, ^64^ Notably, First Nations people lack tailored services despite a threefold higher dementia prevalence among those aged 60 and over in urban and regional areas compared to the national average.^53^^,^^65^ Initiatives such as Koori Dementia Care Project strive to address these gaps.^66^^,^^67^ While technology and telehealth advancements have improved service access for individuals in more remote regions, a persistent obstacle lies in the initial availability of information on how to access these services.^68^, ^69^, ^70^ Financial constraints continue to be a barrier, impacting both individuals in regional areas and those from low socioeconomic backgrounds.^70^

### Comparison of levels of service access for dementia post-diagnosis care

Table 1 provides a snapshot of post-diagnosis service access levels across the six countries. Feigin et al.^71^ offer a framework for levels of access to global neurological services outlining three levels of services: Minimal, Essential, and Advanced. For the purpose of this paper, we modified the framework taking into account varying definitions of post-diagnosis dementia services^1^^,^^3^ to ensure relevant services are included in the country comparison. We also reduced the three levels to two: Essential and Advanced. In the absence of national data or reliable literature on this topic that can be used consistently across those six countries, the table was completed by the authors and their personal communications with other experts in their own country where necessary. Consensus was achieved through multiple discussions and consultations with other experts in the field in each of the countries included in this paper.

**Table 1:** Levels of access for dementia post-diagnosis care and support in the region.

Access to Essential services for dementia care	Countries					
	Lao People's Democratic Republic	Solomon Islands	Philippines	People's Republic of China	South Korea	Australia
**Access to Essential services for dementia care**						
Nurses and other-care workers in the community	**+/−**	**+/−**	**+/−**	**+/−**	**+**	**+**
Primary care doctors (General Practitioners)	**+/−**	**+/−**	**+/−**	**+/−**	**+**	**+**
Essential pharmacological treatments (e.g., cholinesterase inhibitors)	**–**	**–**	**–**	**+/−**	**+**	**+**
Basic preventive and rehabilitation services (hospital outpatient clinics, rehabilitation services or community centre)	**+/−**	**+/−**	**+/−**	**+/−**	**+**	**+**
Care services including personal care provided in local communities (home care) without coordination across defined geographical regions	**–**	**–**	**+/−**	**+/−**	**+**	**+**
Neurologists, geriatricians, psycho-geriatricians	**+/−**	**–**	**+/−**	**+/−**	**+**	**+**
Multidisciplinary memory clinics	**–**	**–**	**+/−**	**+/−**	**+/−**	**+/−**
Neuropsychology, nursing and allied health dementia specialists	**–**	**–**	**–**	**+/−**	**+**	**+**
Respite care or day centre services	**–**	**–**	**−/+**	**+/−**	**+**	**+**
Education and psychological support for people with dementia and family carers	**–**	**–**	**−/+**	**+/−**	**+**	**+**
**Access to Advanced services for dementia care**						
Neurologists with disease-specific expertise	**–**	**–**	**+/−**	**+/−**	**+/−**	**+/−**
Advanced laboratory tests, including genetic testing, and monitoring of therapeutic drugs	**–**	**–**	**+/−**	**+/−**	**+/−**	**+/−**
Advanced neuroimaging tests (e.g. CT, MRI, PET)	**+/−**	**–**	**+/−**	**+/−**	**+/−**	**+/−**
Disease modifying pharmacological treatments (e.g., monoclonal antibody therapies)	**–**	**–**	**–**	**–**	**–**	**–**
Individual support workers for care navigation, support and coordination	**–**	**–**	**–**	**–**	**–**	**–**
Specialist services for behaviours and psychological symptoms of dementia	**–**	**–**	**–**	**+/−**	**+/−**	**+**
Specialist rehabilitation therapy, including cognitive therapy	**–**	**–**	**–**	**+/−**	**+/−**	**+/−**
Community based dementia rehabilitation programmes	**–**	**–**	**–**	**–**	**–**	**–**
Fully coordinated and multidisciplinary neurological/psychiatric/geriatric services across geographically discrete regions	**–**	**–**	**–**	**–**	**–**	**–**

*Note:* ‘-’ No service available; ‘+/−’ Variable service available; ‘+’ Service commonly available.

Due to the lack of national-level data/evidence available in most countries included in this paper, the accessibility of services in this table is based on the authors' expertise and experience in the field, as well as the review provided in each country's vignette.

As shown in Table 1, two lower middle-income countries in this comparison (Lao PDR, Solomon Islands) have minimal level services in dementia post-diagnosis care, as they have variable or limited access to essential care practitioners and specialists, community support and preventive and rehabilitation services. Australia and South Korea appear to have post-diagnosis dementia services across Essential and Advanced levels, but primarily in urban/suburban locations.

## Discussion

The vignettes and service level comparison of the six selected Western-Pacific countries have highlighted that system level post-diagnosis dementia care is highly variable depending on different socio-economic developments. Notably, regardless of economic status, one challenge facing post-diagnosis dementia care is geographical. In countries like the People's Republic of China and Australia, a large geographical size poses logistical challenges, while in countries like the Solomon Islands or the Philippines, multiple small islands create difficulty accessing necessary services for people living with dementia and their families.

We summarise other challenges under three broad themes: logistical/infrastructure and models, societal/cultural, and educational/training.

### Logistical/infrastructure and models

With the exception of the People's Republic of China, few dementia peak bodies, or country-wide policies, exist that can champion the cause of dementia in ***the*** low- and middle-income countries ***included in this Viewpoint***. As such, dementia care generally comes under general healthcare provision (Solomon Islands, Lao PDR) or mental health (the Philippines). This may be in part because dementia is not recognised as a health priority despite population ageing in these countries. This issue is likely to be exacerbated by limited health funding and focus on other health priorities (e.g., immunisation and communicable diseases). Notably, the Philippines has begun developing a nationwide approach to improve dementia awareness, early diagnosis and treatment and care pathways.^30^ The absence of a national dementia strategy in those countries clearly signals first the need for building a coordinated and intersectoral or integrated national framework and policy.

The urban/rural divide drives considerable disparities in access to services regardless of economic status. Indeed, access to specialist services with the necessary diagnostic investigations are available predominantly in large urban centres or in more developed regions (e.g., the People's Republic of China). Another important point is the need for a diagnostic and post-diagnosis care system that provides a national direction and locally tailored centres, such as the one in South Korea. Such a ‘hub and spoke’ model that considers the local environment and resources available would provide a flexible framework to deploy resources effectively and promote equity in access to services. Notably, Australia's national approach to behaviour assessment and management as post-diagnosis care beyond hospital or specialist physicians' care,^60^ is unique among the countries included in this Viewpoint.

As shown in the vignettes from Lao PDR and Solomon Islands, post-diagnosis dementia care is largely limited to the hospital setting. Such focus on hospital or acute-care led dementia care can be a major barrier to the implementation of evidence based post-diagnosis dementia services that have been largely modelled for the community.^3^^,^^4^ Establishing and maintaining intersectoral partnerships between hospital, primary care and community care settings is likely to strengthen post-diagnosis services for people living with dementia and their family carers.^72^ A lack of care coordination and integration, however, remains a major issue in many other countries, beyond the six countries included in this paper.^72^

One of the key recommendations for post-diagnosis care is implementing a case management model, where an appropriately trained professional provides care coordination and links people with dementia to multidisciplinary teams.^4^ This approach can be particularly challenging in low- and middle-income countries lacking allied health workforce and services. As highlighted in several international reports,^1^^,^^4^^,^^5^ balancing skilled workforce demand and supply is crucial in quality dementia post-diagnosis care. Understanding dementia workforce availability, service availability, and gaps across a pre- and post-diagnosis spectrum in those countries is urgently needed. This could be achieved through, for example, service mapping and environmental scanning studies.

Furthermore, the interconnectedness between dementia risk reduction, diagnosis, treatment, and management is crucial in successfully improving post-diagnosis care. Rates of dementia diagnosis vary dramatically depending on country income, being about a third in low- and middle-income (21%) compared to high-income (58%) countries.^1^ The introduction of new biomarkers for dementia will speed up and improve diagnostic efficiency and the development of disease-modifying therapies that will require additional services and infrastructure. Rigorous and comprehensive national dementia planning will be ever more important globally to ensure equitable access.

### Societal and cultural attitudes and behaviours

A significant roadblock to dementia care comes from how dementia is perceived in the general population. The 2019 global survey of dementia across 155 countries showed that most of the survey participants still believed dementia to be part of normal ageing.^73^ Importantly, this issue is not limited to the low- and middle-income countries. A recent survey conducted by the Australian government showed that over 20% of people interviewed believed that dementia was part of the normal ageing process and failed to recognised dementia as a brain disease.^74^ Integrated post-diagnosis dementia care will only be possible once dementia is recognised as a neurodegenerative brain condition that is not part of the normal ageing process.

It is increasingly accepted, albeit slowly, that living well with dementia is possible and should be promoted. A bourgeoning volume of research concerning non-pharmacological interventions, underpinned by principles of person-centred care, has shown strong support for this recent trend. This reframing of dementia has also been influenced by a growing voice of advocates who have lived experience with dementia, with a strong emphasis on human rights approach: right to live independently, and right to access care and services needed. Focusing on the person's abilities, beyond their disabilities, and optimising their health and wellbeing through participation in their daily, physical, social, and community activities, can lead to improved mental, emotional, cognitive, and physical function, self-care abilities, as well as enhanced carer wellbeing, and potentially reduce unplanned hospitalisations, falls, and institutionalisations.^5^

The WHO's Rehabilitation 2030 campaign calls for action to optimise the power and potential of non-pharmacological interventions under rehabilitation.^5^ Along with the national dementia strategy, having dementia rehabilitation as part of Universal Health Coverage provides countries with an important direction for the health and wellbeing of people living with dementia and their informal carers. Stigma-stricken conditions, such as dementia, suffer from therapeutic nihilism and misconception, whereby a diagnosis is believed to be associated with a death sentence with no option to live well after a diagnosis of dementia. These societal and cultural misconceptions appear accentuated in low- and middle-income countries.^73^ The Philippines' and the People's Republic of China's recent introduction of a national dementia action plan shows promising progress and provides impetus for other countries in the region. Importantly, positive narrative campaigns without appropriate diagnostic and post-diagnosis services in place have limited benefits. South Korea's national approach to fight against dementia has achieved positive changes in dementia care in a relatively short-time period. Their public campaigning aligned well with policy directions and service developments with a concerted effort across schools, health services, community organisations as well as governments at scale, played a key role.4 Australia has also made a growing investment in improving dementia literacy and stigma reduction through dementia friendly communities and public education and campaign activities^60^; however, access to appropriate post-diagnosis dementia service availability is lagging, especially in rural and remote communities.

### Educational/training

Provision of relevant care following a dementia diagnosis is only possible if information about the disease exists. Prevalence data are necessary to inform the development of services and infrastructure. In parallel, information is needed on the uptake, outcomes, or impact of these services (e.g., in the Philippines), which will, in turn, inform national policies. Equitable access to dementia care will also need to consider the geographical challenges outlined above (e.g., the People's Republic of China, Australia, the Philippines, Solomon Islands).

Continuing education of healthcare professionals and general population awareness campaigns remain foundational aspects of dementia care. Continuing education helps identify knowledge gaps at all stages of the dementia journey (diagnosis, management, treatment, rehabilitation, palliative care), while awareness campaigns increase services utilisation and promote dementia risk reduction. Workforce capacity building across all care settings and among professional and non-professional workers is a crucial element of pharmacological and non-pharmacological post-diagnosis care.^61^ The WHO's Package of Interventions for Rehabilitation (Dementia)^75^ provides guidance for appropriate training and education for those care practitioners involved in various types of dementia rehabilitation. In Australia, a federally funded dementia training program offers opportunity for workforce capacity building across diverse workforce and care settings.^59^ However, such nationwide education and training on the care of people living with dementia for almost a decade has not been fully embraced by those who most need training,^76^ suggesting that such training programs without appropriate regulatory requirements for skilled workforce may not reach their full potential.

## Conclusion

Improved dementia care is associated with higher quality of life, increased lifespan, and a greater number of years lived with the disease, which in turn is associated with higher socioeconomic burden. Innovative solutions for post-diagnosis dementia care are likely to emerge from the use of novel technologies. The World Alzheimer Report 2022 *Life after Diagnosis: Navigating Treatment, Care and Support,**^4^* provides a comprehensive review of the need for and impact of post-diagnosis care, discussing challenges and recommendations for a way forward. For example, a model of care based on ‘hub-and-spoke’ networks (with dedicated regional centres supported by central expertise) is fast becoming an attractive proposition, in light of decreasing costs and faster access to the internet; a flexible model that can be deployed regardless of distances and/or geographical isolation.

We have attempted to paint a picture of post-diagnosis dementia care in selected countries with varying economic developments in the region. This Viewpoint provides an initial step for future research, highlighting the urgent need for research on dementia service needs and gaps in the lower- and middle-income countries in the region.

## Contributors

**Conceptualisation** Yun-Hee Jeon, Olivier Piguet. **Original draft:** Yun-Hee Jeon, David Foxe, Guk-Hee Suh, Huali Wang, Jacqueline C. Dominguez, Rex Maukera, Sengchanh Kounnavong, Olivier Piguet. **Review, edits, and revision:** Yun-Hee Jeon, David Foxe, Guk-Hee Suh, Huali Wang, Jacqueline C. Dominguez, Rex Maukera, Sengchanh Kounnavong, Olivier Piguet.

The authors alone are responsible for the views expressed in this publication and they do not necessarily represent the decisions or policies of their affiliated institutions.

## Editor note

The Lancet Group takes a neutral position with respect to territorial claims in published maps and institutional affiliations.

## Declaration of interests

Y-HJ has received research grants from the National Health and Medical Research Council (NHMRC) and Arcare in Australia and travel support for invited talks from Singapore SingHealth, Northern Ireland Queen's University Belfast, Hong Kong Jockey Club, and Korea National Health Insurance Service: Long-term Care, all outside the submitted work. DF is supported by the Edwards Fund for Dementia Research and has received research grants from the Dementia Australia Research Foundation, outside the work submitted. GHS, JCD, RM, and SK have nothing to declare and no conflict of interest. HW has received research grant from the Science and Technology Innovation 2030 and Beijing Municipal Science and Technology Commission, outside the submitted work. OP is supported in part by NHMRC Leadership Fellowship and has received research grants from the NHMRC and the Australian Research Council, outside the submitted work.
